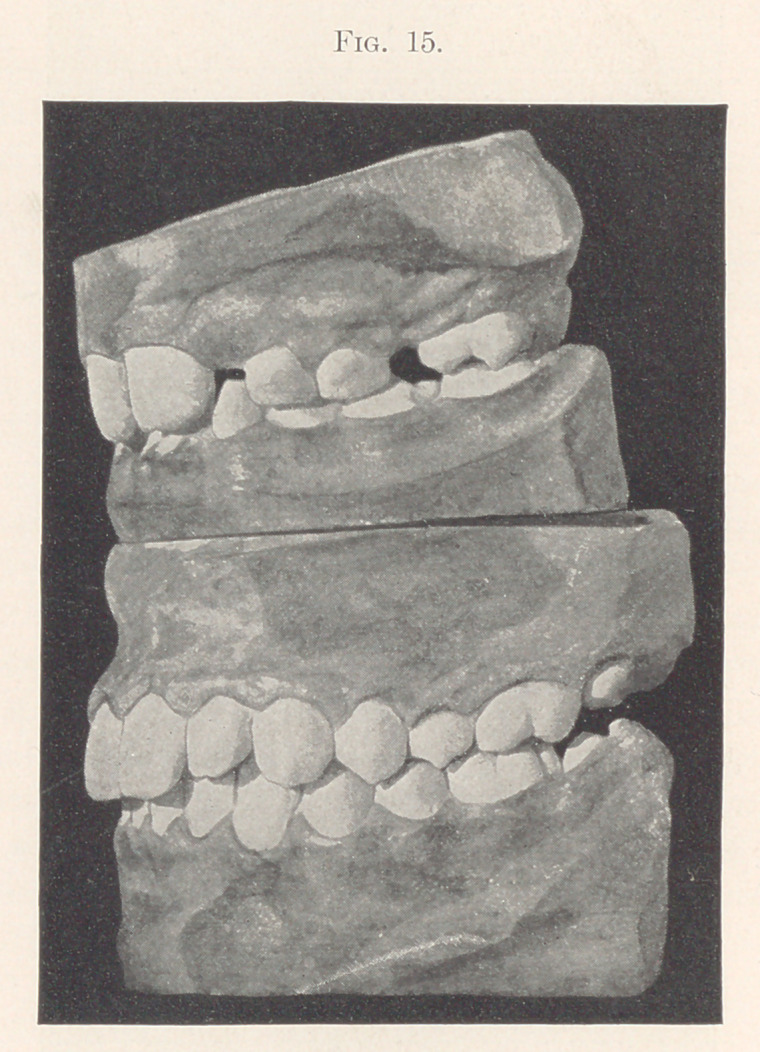# The Principal Molar in Man, and Its Relations to, and Bearings upon, the Other Teeth

**Published:** 1903-08

**Authors:** E. A. Bogue


### International Dental Journal.

Vol. XXIV. August, 1903. No. 8.

### Original Communications.[1]

### THE PRINCIPAL MOLAR IN MAN, AND ITS RELATIONS TO, AND BEARINGS UPON, THE OTHER TEETH.

BY E. A. BOGUE, M.D.

By principal molar I mean the first permanent molar, or what is often called the sixth-year molar.

It gets its start soon after birth. Three to four years after the temporary teeth are all in position, and all in use, this principal molar begins to make its appearance through the gum, generally at about the age of six years. As all present know, it erupts next back of the last temporary molar.

When these principal molars are nearly erupted an impression of all the teeth will indicate, with almost absolute accuracy, if there is to be any serious irregularity among the permanent teeth. This may appear strange, and perhaps incomprehensible, to those who have given the subject of dental dislocations little or no attention, but I think I shall demonstrate to your entire satisfaction the truth of my assertion.

---

[1] The editor and publishers are not responsible for the views of authors of papers published in this department, nor for any claim to novelty, or otherwise, that may be made by them. No papers will be received for this department that have appeared in any other journal published in the country.

I am not proposing to tell you new things, but to recall to your attention old things that you know quite as well as I.

First, then, this principal, or first permanent, molar has in the lower jaw five cusps (though sometimes the fifth is but a rudimentary one) and four in the upper jaw. These cusps are of great consequence to the teeth from the very beginning. They assist in guiding the upper and lower teeth to their proper positions relative to each other, and when these positions have been attained they assist in holding them there. If, by any chance the teeth have assumed improper positions, and the cusps of the upper and lower teeth have interdigitated improperly, they assist in holding the teeth in their improper positions just about as thoroughly.

As these four principal molars erupt, the lower ones being a little in advance of the upper in point of time they should also be one cusp in advance of the upper in point of position; that is to say, the upper molars should sit astride of the outer, or buccal, cusps of the lower molars, but just behind the anterior cusps.

We all know that just room enough is required between the principal molars and the cuspids for two bicuspids. If, therefore, the proper occlusion of these principal molars fails, there is bound to be an irregularity of some kind. If the upper molar is in advance of the lower, there will not be room between that molar and the cuspid for the two bicuspids, and we shall have either an irregularity of the cuspid or of one or both of the bicuspids.

If, on the other hand, the lower molar is too far in advance of the upper, the irregularity will be most manifest among the lower teeth.

I call it the principal molar in deference to the expressions of famous anatomists more than one hundred years ago; Blainville, for example, who is much quoted by Owen; besides, I find the name so appropriate, in view of more modern discoveries, that I think we shall all do well to adopt it.

I wish now to call your attention to Dr. Thompson's picture (Fig. 2), which you will find in Kirk's "American Dentistry."

You notice that the principal molar is, as above stated, almost exactly in the middle of the arch from before backward. You will notice that the principal molar, the second molar, and the wisdom-tooth are three, and that they are all first teeth, notwithstanding they are also permanent teeth, and that they all come into the row of teeth back of the temporary set, and that the addi-

Fig. 1

Fig. 2.

Fig. 3.

Fig. 4.

tions to the skull, to the jaws, and to the alveoli of those jaws necessary to accommodate these teeth all take place between the time of the eruption of the first or principal molar and the eruption of the third molar or wisdom-tooth.

Thus we see that the growth necessary to the completion of the countenance, that growth which we say comes with increased years, that growth upon which depends so much the character of the mature face, is largely dependent upon the presence of these three molars for such development. Now turning to the forward lateral portion of the dental arch we find again three teeth,—two bicuspids and the cuspid. These complete the line and the curve from before backward. Forward of the cuspid stand the two incisors, which complete the arches of the teeth.

Nearly all of the growth that has taken place in both the jaws since the eruption of the principal molar, at about six years of age, has taken place posteriorly to, and above, this molar.

This growth has occurred in the interior of the skull and leaves the anterior face of this molar as the middle of the dental arches.

The lower model in Fig. 4 shows as nearly a perfect set of teeth, both in form and arrangement, as we shall often find. Not only are these teeth theoretically perfect, or nearly so, in position, but I was not able to find the least trace of decay at any point. The lady to whom they belonged was probably about thirty years of age. The upper model shows us a case in which the upper principal molars developed a very little farther forward than they should have done. The result is a slight prominence given to the cuspids, imparting to one of the mildest and gentlest of faces a sort of fierce look when the person shows the teeth.

There is also a depression at the incisal region that accentuates still further the prominent cuspids.

The principal molar is, indeed, the key-stone of the dental arch, and when it is realized that every member of that arch is of a size exactly appropriate to the perfect arch, it is seen that any variation in the position of this key-stone causes a variation in the entire arch.

Dr. Talbot, in his "Irregularities of the Teeth," fourth edition, page 63, says, "If the teeth antagonize uniformly, the arch will enlarge around evenly. If the molars are fixed, the alveolar process will expand anteriorly." Also, "The position and shape of the processes and their relation to each other are governed entirely by the shape and size of the teeth and roots, and not by the shape of the jaw-bone proper."

Please understand that I have no quarrel with the doctrine of development. On the contrary, I recognize therein the Creator's method of creation, but I have a very decided objection to the doctrine that a six-foot Irish father, strong and vigorous, can give his teeth to the jaw of a five-foot French mother, frail and thin, and can combine these two qualities in the progeny of those two parents. That is not the way that nature works, and for us to attempt to evade our responsibility as scientific men, practising for the children a calling that has much to do with all their future lives, both as regards health and appearance and powers of expression, whether vocally or by nobility of countenance, is beneath our dignity as members of one of the learned professions.

When Nature builds a set of teeth she builds a jaw to correspond, and if that jaw comes to maturity and the teeth are developed and find their proper positions along nature's lines without accident or improper meddling, it will be found that not only is the jaw large enough for the teeth, but also that under these conditions whatever facilitates the natural and proper development of that jaw and the natural and proper placing of those teeth into the most perfect dental arches also facilitates the development of the other facial bones. The ethmoid, sphenoid, and palate bones, as well, have developed nicely, and the sense of hearing and smelling and the power of mastication, as well as enunciation, are all promoted by this normal dental arch development.

In order to guide the teeth in their development into proper positions, it is evident that it is necessary to know precisely what those proper and natural positions are. Hence it is necessary for us to study carefully the arrangement of a good many sets of thirty-two natural teeth. There is, of course, no absolutely perfect and typical set of teeth, any more than any perfect part of the body. We should study these complete sets from a period pretty well along in life, when the teeth shall have suffered the wear and tear of forty years or more, and thence backward through the various stages of accident and development to the very beginning. Upon such a study as this will depend our ability to guide and to preserve in good order the teeth of our patients from infancy to old age. If we do anything to disturb the natural ideal position of the teeth we interfere with nature's process of arrangement, as well as with that of self-cleansing. If we interfere with the

Fig. 5.

Fig. 6.

Fig. 7.

Fig. 8.

Fig. 9.

Fig. 10.

Fig. 11.

Fig. 12.

cleansing, we promote decay, for a clean tooth never decays; and when we permit the teeth to go astray because of what we call accidental circumstances that occur during the processes of formation and development of the permanent teeth, we contribute to their loss.

We will now follow through a few cases illustrating certain conditions that are frequently found in practice.

Fig. 5 shows the first permanent molars above and below in perfect occlusion, and presupposes room enough forward of these teeth for all the remaining teeth to erupt with perfect regularity.

Please remember that an amputation or an extraction is no cure for a dislocation of any member. It is only an evasion of the difficulty, and a substitution of one of *our* mistakes for a mistake of Nature's, to the greater detriment of the patient.

The models exhibited in Fig. 6 both show in a marked degree the important office of the principal molars, as shown in the previous model, to sustain the jaws during the shedding of the temporary teeth and their replacement by the permanent ones.

The lower model (Fig. 6) shows the irregularity produced by lack of room, owing to a dislocation of the upper molar. Imagine what would happen to these jaws were the principal molars to be extracted at this age,—about ten years.

The two cases shown in Fig. 7 exemplify a condition of the principal upper molar that is not quite accurate, and would *a priori* indicate an irregularity of the molar and bicuspid teeth, and possibly of the cuspids, owing to the upper principal molar being one cusp in advance of what it ought to be. As an actual fact you see that the cuspid in one case and the bicuspid in the other have erupted out of their proper place and are unable to resume their places in the arch because the upper molar is too far forward and does not leave enough room for the other permanent teeth in front of the upper molars; hence we find in each case a tooth crowded out of position.[1]

---

[1] It is probable that the somewhat more anterior position of the molar, as well as that of one or both of the bicuspids, is secondary, and the effect of a too early shedding of the posterior premolar, or a delay in the eruption of the canine. Hence it may be better, by all means safer, to say the principal molar *assumed* a wrong position, not erupted in such.

I am indebted to Dr. A. Hrdlicka, Curator of Physical Anthropology in the National Museum at Washington, Smithsonian Institution, for the above note, as well as for a number of suggestions which have increased the accuracy of this communication.—E. A. B.

The two cases shown in Fig. 8 represent the eruption of the *lower* principal molar forward of what it ought to be, and a consequent diminution of room in the *lower jaw,* with the effect of throwing the somewhat belated first bicuspid far out of line in the upper model, while the cuspid is rotated upon itself and thrown inward. In the lower model the impulse forward has been extended to the cuspids and incisors, giving the young lady a somewhat square, unseemly front part of the lower jaw.

Figs. 9 and 10 show the model of a boy eight years old in May, 1900. It exhibits the left upper molar somewhat more than a cusp in advance of where it should be relative to the lower molar. In this case the second upper temporary molar had fallen out of its own accord, and the permanent upper molar was found in the position which it occupies on the model when this impression was taken. A fixture was put on to press this upper molar back to place. About that time the bicuspids began to erupt, and presented the appearance shown in the next two slides (Figs. 11 and 12).

Upon succeeding in getting the molar back to its proper position, the bicuspids on each side of the mouth were easily drawn into their places in about ten days. Retaining-plates were kept in position until the cusping of the upper and lower teeth with each other was sufficient to hold them in place so that the heavy pressure exerted by the tongue on one side, and lighter but continuous pressure of lips and cheeks on the outer sides, completed the oblong form of the two arches, and the result is what we see in the model of the finished case, which I present to you also (Figs. 13 and 14).

Fig. 15 contrasts the two models before and after the operations for correcting the malocclusion.

The point to which your attention has been especially though indirectly called during all this description is, that the removal of one or more teeth from either of the dental arches does not and cannot correct any irregularity of any kind. *Nor does it aid in correcting such irregularity.* I beg your careful consideration of this statement after reading it over. I can conceive of cases of irregularity having been neglected until maturity that might be somewhat benefited by the removal of one tooth, but I cannot conceive of any case where the extraction of four teeth could ever be of sufficient benefit to compensate for the extra mischief done; nor is it ever good practice to extract permanent teeth from children or young persons. All necessary corrections of irregularities

Fig. 13.

Fig. 14.

Fig. 15.

can be done more promptly and more easily for young people with all the teeth in place.

When this cusping of the upper with the lower teeth is carefully considered, it is recognized that the loss of even as much material as would be removed by a thin file passed between two teeth works a damage difficult, if not impossible, to wholly repair. On the other hand, teeth that are irregularly situated, that is, dis-located, submit to being drawn into their proper places, with the remaining teeth all in position to assist in holding them there, much more readily than they submit to be pulled into places where they did not originally belong. In the former case the alveolus bends a good deal, submits to absorption somewhat, and the teeth are brought into their proper arches and their proper contact with each other, and are held by natural forces, prominent among which is their proper cusping with the antagonizing teeth of the opposite jaw. In the latter case, when teeth have been extracted to be regulated, the teeth are forced against a wall of alveolus that was designed to hold them for life. The force required and the time consumed to draw them into improper positions is so much greater than what would have been required had all the teeth remained in their proper relations with each other, that from two to three years are frequently consumed in obtaining only a fair condition of regularity, with no proper occlusion at all and no possibility of properly grinding the food which is taken.

I now call your attention to the main lesson that we may draw from all which has preceded.

We may at about seven years of age, perhaps even sooner than that, accurately foretell whether or not any irregularity is impending among the molar and bicuspid teeth of a child.

And we may, as soon as the first molar teeth are developed enough to attach rings to them, or sometimes to tie a wedge between them and the temporary molars, correct such irregularities surely and painlessly.

The upper model in Fig. 16 shows the mouth at six years of age, with the first permanent molars, erupted and slightly in contact, occupying their normal position,—namely, the lower molar one cusp in advance of the upper. The temporary teeth are in their places, and there is nothing especially noticeable, certainly nothing requiring attention among the teeth. Ten months later the lower model was taken. Here we find the upper molar has advanced one cusp forward of the position it ought to occupy. The four temporary teeth above and below are still in place, but the upper ones have been pushed forward of the position which they occupied ten months previously. The permanent central incisors have erupted and stand at a V-shaped angle, nearly if not quite three-eighths of an inch forward of where they should be. Now the question arises, How did this extreme prognathism of the upper jaw take place in so short a time, and why, and what should be done to correct it, and when should it be done?

Fig. 17 is the front view of the same mouth, taken at the same dates as the previous slide, showing very clearly the good occlusion of the temporary teeth previous to the growth of the permanent molars and the malocclusion of these same teeth when the permanent molars finally erupted to the length shown in the picture. These models show to me a mouth-breather, with a slight diminution of width in the region of the cuspids, which made it disagreeable to the child to close the teeth naturally, so she acquired the habit of closing to one side to get rest.

During this period the permanent upper molars grew down and failed of proper occlusion with the lower ones, and so acquired a position in advance of what they should have had. This, unless corrected, will surely cause irregularity among the teeth forward of these molars, because of insufficient room for them to erupt into their proper positions. My own conviction is, and has been for some time, that if the principal molars can be got into their proper relative position at or shortly after the time of their eruption no serious irregularity will ever occur to any of the grinding teeth. Irregularities arising in the incisal region are extremely easy of correction providing the principal molars are in their proper positions.

## Figures and Tables

**Fig. 1 f1:**
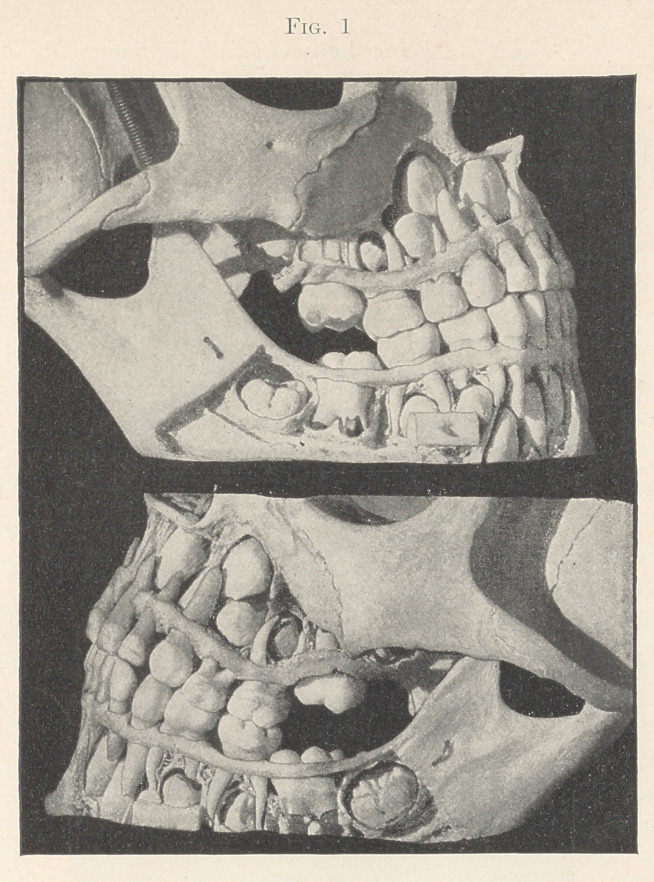


**Fig. 2. f2:**
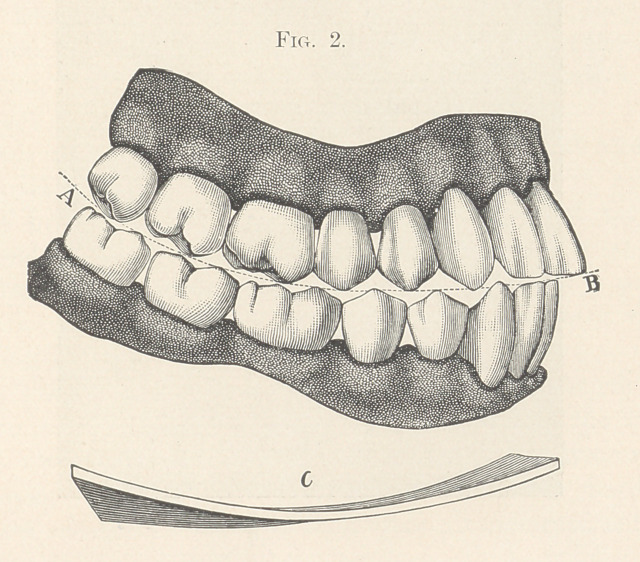


**Fig. 3. f3:**
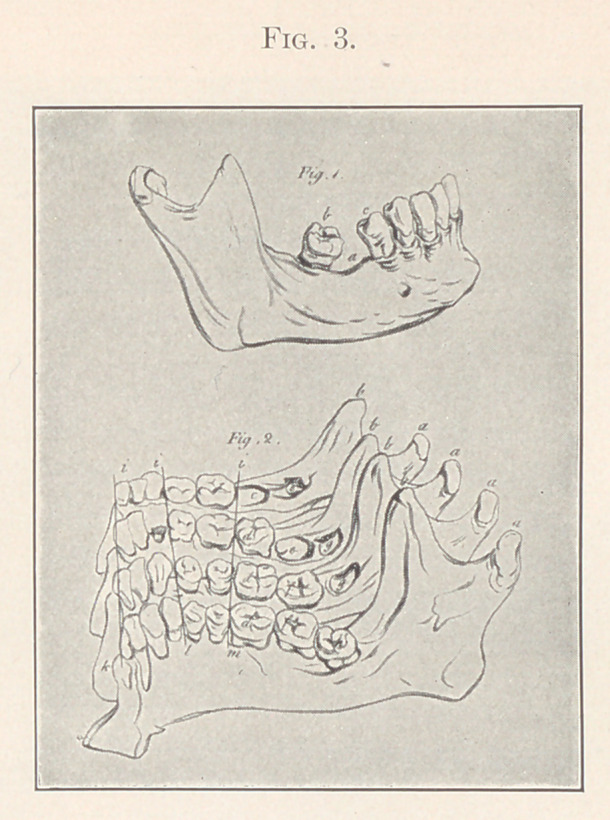


**Fig. 4. f4:**
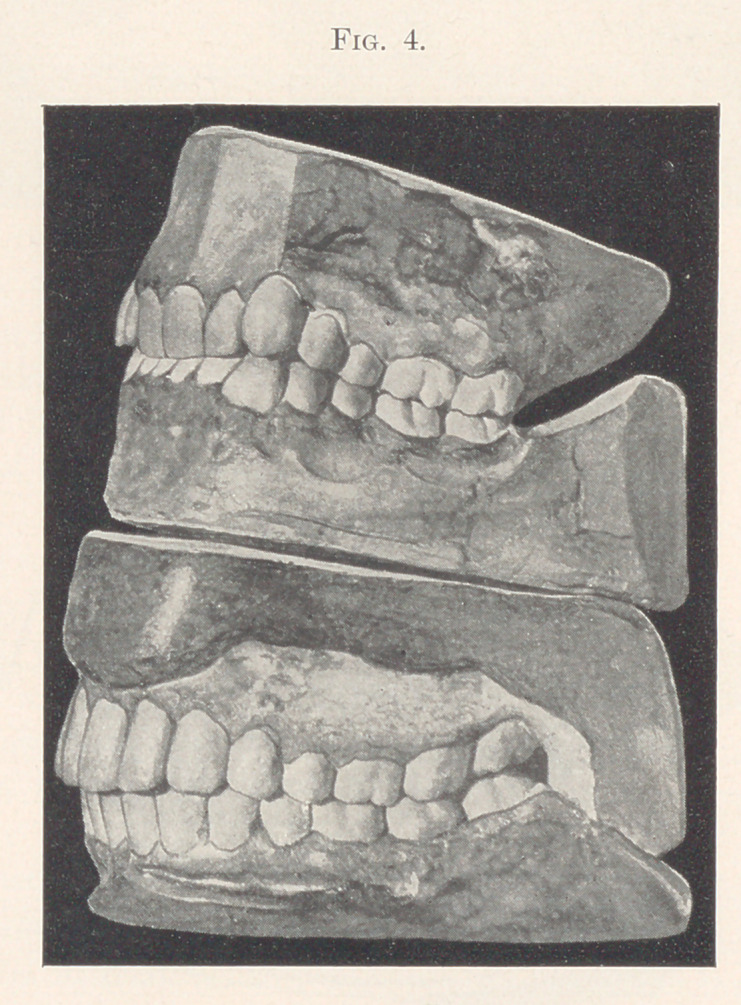


**Fig. 5. f5:**
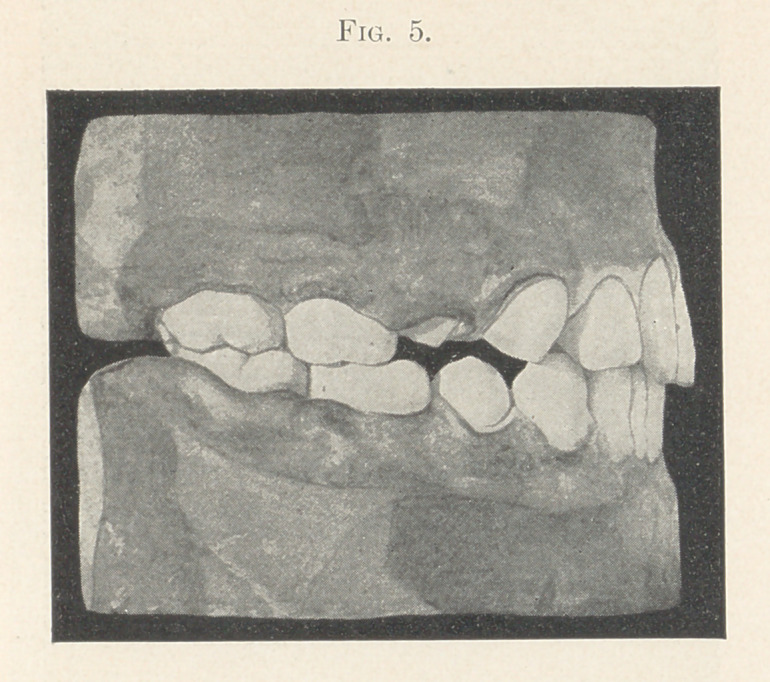


**Fig. 6. f6:**
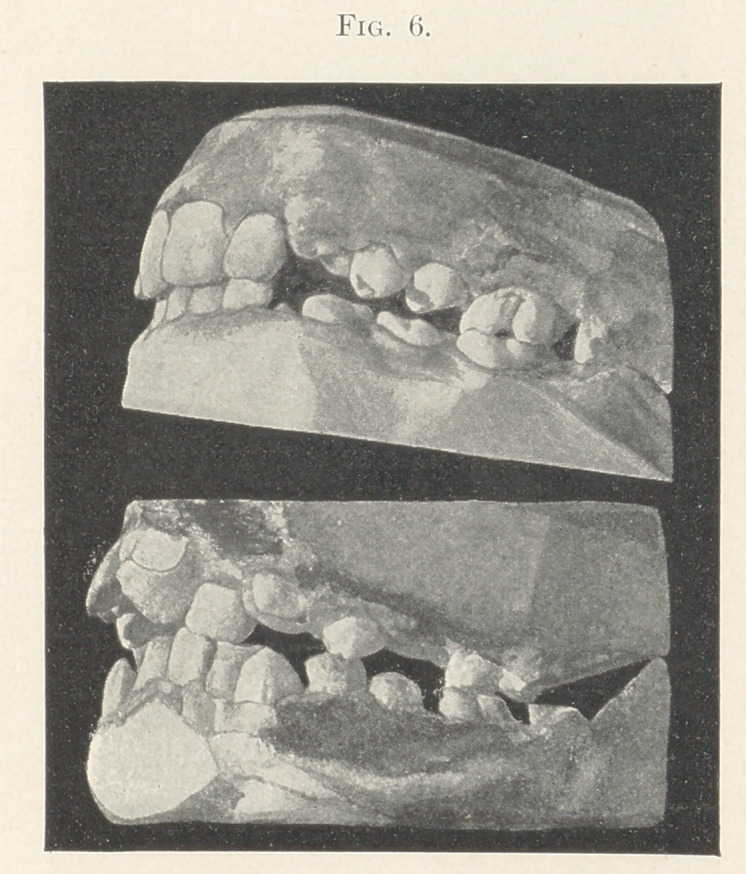


**Fig. 7. f7:**
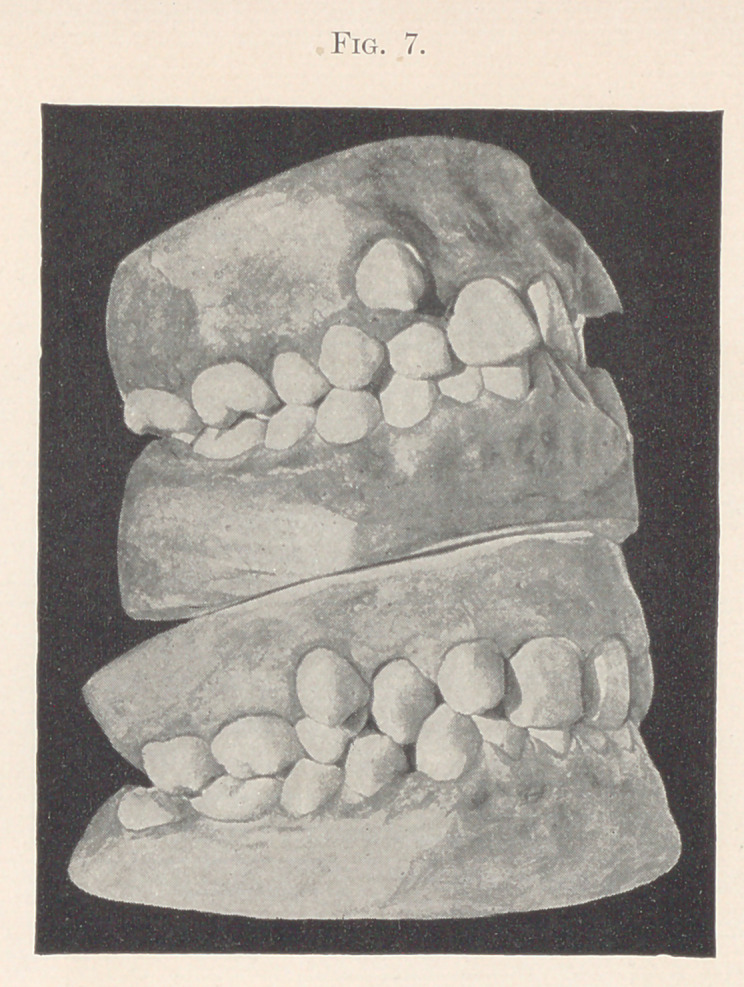


**Fig. 8. f8:**
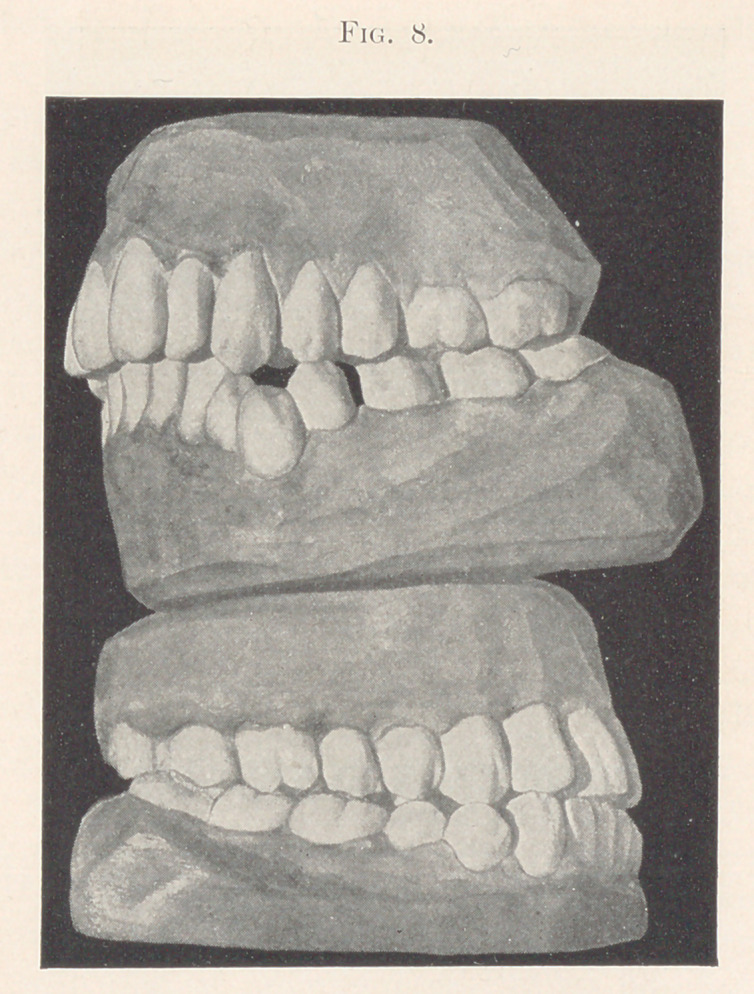


**Fig. 9. f9:**
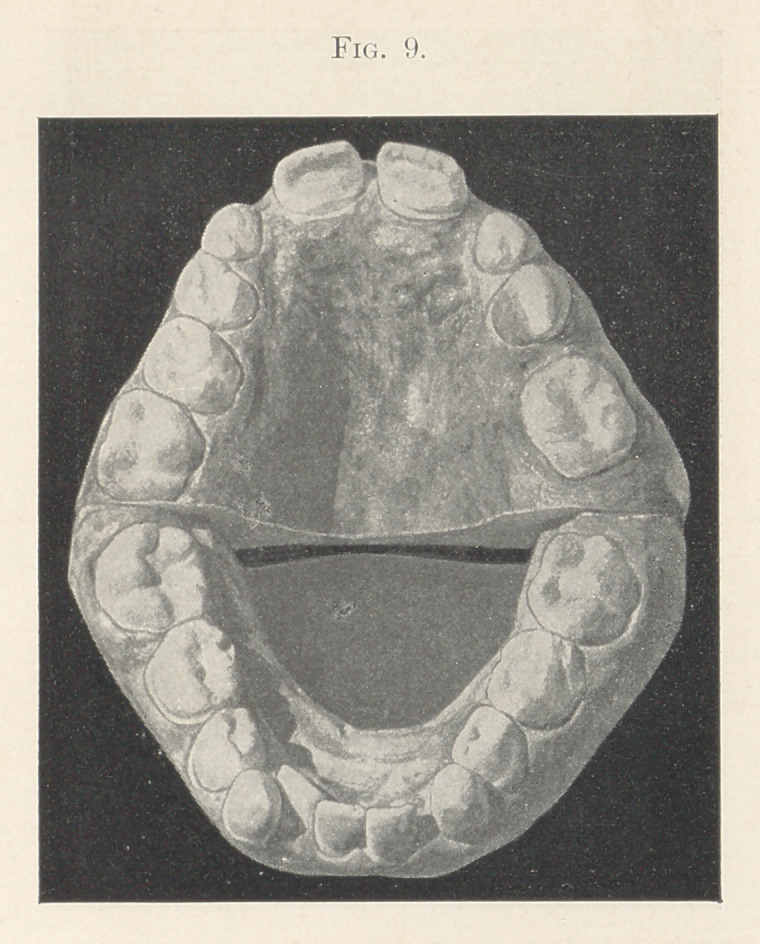


**Fig. 10. f10:**
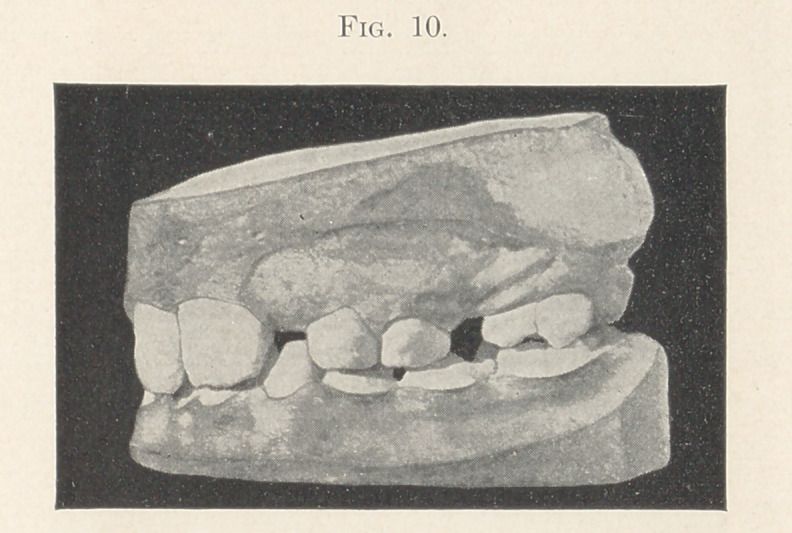


**Fig. 11. f11:**
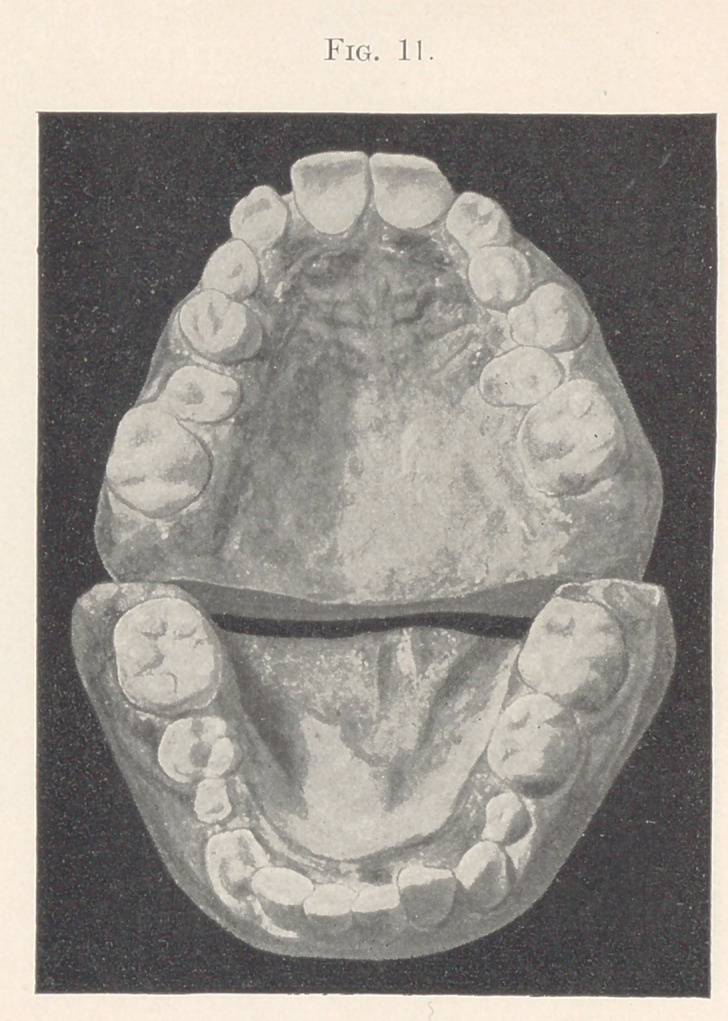


**Fig. 12. f12:**
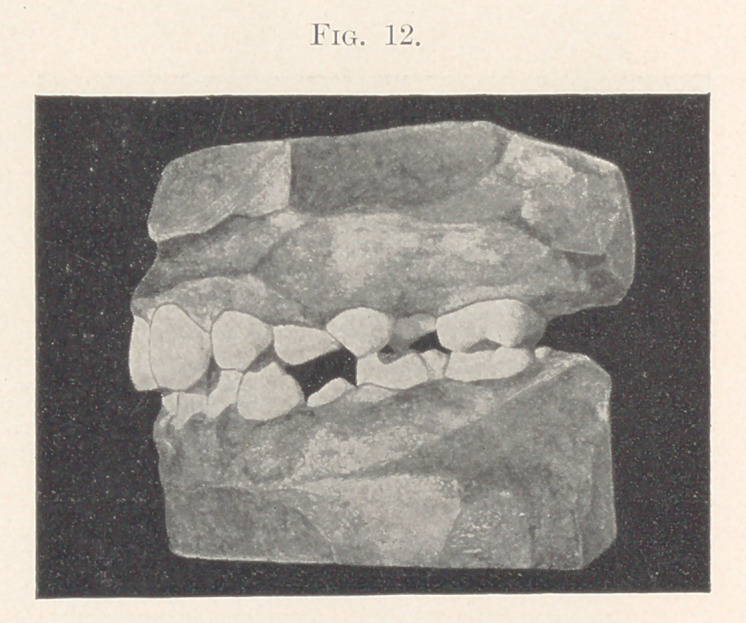


**Fig. 13. f13:**
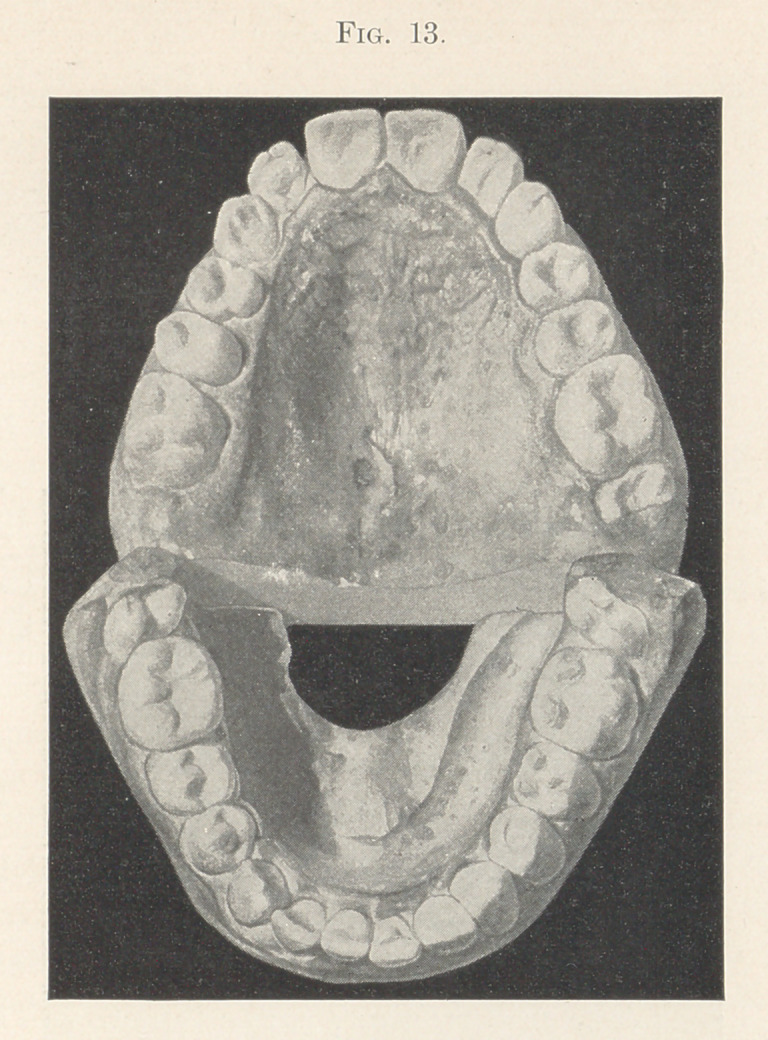


**Fig. 14. f14:**
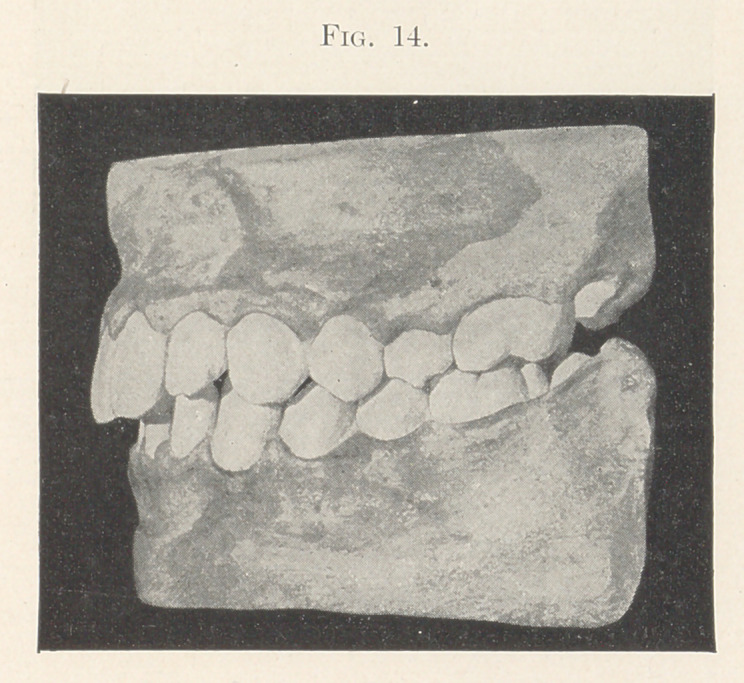


**Fig. 15. f15:**